# Litigation in paediatric urology within the National Health Service (NHS) England: an analysis of costs and causes

**DOI:** 10.1007/s00345-026-06362-9

**Published:** 2026-03-28

**Authors:** Panagiotis Nikolinakos, Katie McComb, Marina Maria Antonaraki, Ivo Donkov, Nadir I. Osman, Karl H. Pang

**Affiliations:** 1 Department of Urology, Chelsea and Westminster Hospital NHS Foundation Trust, London, UK; 2 Department of Pediatric Surgery, School of Medicine, Attikon University Hospital, National and Kapodistrian University of Athens, Athens, 12462 Greece; 3 School of Medicine, National and Kapodistrian University of Athens, Athens, Greece; 4 Department of Urology, Sheffield Teaching Hospitals NHS Foundation Trust, Sheffield, UK; 5 Division of Surgery and Interventional Science, University College London, London, UK

**Keywords:** Paediatric urology, Paediatric surgery, Litigation, Complaint, NHS England, Malpractice

## Abstract

**Objective:**

To provide a national evaluation of contemporary litigation trends within Paediatric Urology within National Health Service (NHS) England by examining long-term trends, the financial burden and the underlying causes of successful claims over the last 19 years.

**Materials and methods:**

Litigation data were obtained from NHS Resolution through a Freedom of Information (FOI) Act 2000 request, from 2006/2007 to 2024/2025. For all paediatric urology cases (patients < 18 years), information was collected on the total number of claims, the number of which were successful, and the associated costs of damages and legal fees. Successful claims were reviewed according to the recorded primary cause and the primary injury sustained.

**Results:**

In total, 216 litigation claims were submitted in Paediatric Urology during the study period, of which 134 (62%) were successful. Damages paid amounted to £5.32 million. The number of claims rose 3.6-fold over the 19-year-period. Damages increased 2.2-fold, from £195,750 in 2010/2011 to £441,410 in 2024/2025. The leading causes of successful claims were failure or delay in diagnosis (29.1%, 39/134), failure or delay in treatment (24.6%, 33/134), and inappropriate treatment (4.4%, 6/134). Operative factors accounted for 9.7% (13/134), while non-operative causes represented 66.41% (89/134). The most common primary injuries were removal of a testicle (36.5%, 49/134), additional or unnecessary procedures (17.1%, 23/134), unnecessary pain (8.9%, 12/134), and renal damage or failure (6.2%, 9/134).

**Conclusion:**

Both total and successful Paediatric Urology claims have progressively risen steadily across NHS England. Most stem from non-operative issues potentially influenced by extended waiting times. Improving clinician education, promoting learning from avoidable incidents, and ongoing monitoring are essential to mitigate risks, improve patient outcomes, and reduce medicolegal costs.

## Introduction

Litigation arising from clinical care continues to place considerable burden on healthcare systems, including the National Health Service (NHS). Surgical specialties such as Paediatric Urology which frequently involve complex clinical decision making, and high-risk interventions remain among those exposed to litigation in England [1, 2]. In 2023/2024 NHS Resolution reported expenditure of approximately £2.8 billion across its clinical negligence indemnity schemes, representing a continued year-on-year increase from £2.64 billion in the preceding financial year [3]. Beyond illustrating the increasing financial burden, national litigation data also provide valuable insight into contributing factors associated with avoidable patient harm.

NHS Resolution, previously known as the NHS Litigation Authority, is a body of the Department of Health and Social Care that supports the NHS in the fair resolution of concerns and disputes, while promoting organisational learning to improve patient safety [4]. Its strategic approach emphasises “resolution through collaboration” underpinned by fairness and the use of data and insights to drive improvement [4].

A detailed understanding of the drivers and financial implications of litigation within Paediatric Urology is therefore essential for clinicians, healthcare organisations, and policymakers seeking to enhance patient care while safeguarding NHS resources. This study aimed to analyse litigation data provided by NHS Resolution to examine trends and underlying causes of claims relating to Paediatric Urology within NHS England over a 19-year-period.

## Materials and methods

Litigation data were obtained from NHS Resolution through a request made under the Freedom of Information (FOI) Act 2000 [5]. Information relating specifically to the specialty of Paediatric Urology (patients < 18 years) were supplied by NHS Resolution in Portable Document Format (PDF) and covered the period from 2006/2007 up to 2024/2025. The dataset was current as of 25 November 2025. Paediatric urological claims were defined as those where the specialty field was coded as urology and the paediatric flag indicated age < 18 at the time of the incident. The data were provided separately from adult Urology and paediatric surgery datasets. Supplementary keywords or condition-based filters were not available. The authors did not reassign or reclassify specialty categories.

Data were reported on an annual financial year basis across a 19-year period, from 2006 to 2025. This included the total number of Paediatric Urology claims, compensation paid in damages, and associated legal costs incurred by both claimants and the NHS. Data on successful claims were further categorised by their primary cause across the study period. For each category, details were recorded on the type of issue (e.g. failure/delay in treatment, inappropriate treatment etc.) how often it occurred, and the total damages awarded. All data were collated and analysed descriptively and plotted on line graphs using Microsoft Excel for Mac (version 16.103.2). All categorisations of primary cause and injury were assigned by NHS Resolution and retained unchanged in the analysis. The data from NHS Resolution did not stratify into type of urological procedure.

In line with Data Protection requirements, categories containing fewer than 5 claims were masked with a “#” as the likelihood exists that individuals who are the subject of this information may be identified either from this information alone or in combination with other available information. Furthermore, some total values may have also been approximated to prevent masked data from being inferred through reverse calculation.

NHS Resolution defined a successful claim as one resulting in payment of damages to the claimant, either through settlement, court judgement, or agreement including a periodical payment order (PPO) [6]. Claims classified as unsuccessful include those defended successfully, discontinued or closed without payment [6].

It should be noted that paediatric care of genitourinary conditions within NHS England may be delivered across multiple specialties, including Paediatric Surgery, and in some settings, adult Urology. The FOI extract represents claims coded as “Urology” with a paediatric flag (< 18 years) within NHS Resolution’s database and may therefore not capture all paediatric genitourinary-related claims recorded under other specialty codes.

## Results

Across the financial years 2006/2007 to 2024/2025 a total of 216 litigation claims (range 5–20 claims per year) were notified to the NHS Resolution. Of these 134 (62%) were successful (range 6–12 claims per year). The cumulative financial burden for successful claims over the 19-year period was £10.23 million, comprising of £5.32 million (range £0.063–1.46 million per year) in damages, £0.79 million in NHS legal costs, and £4.11 million in claimant legal costs. This corresponds to an average total cost of approximately £76,000 per successful claim.

### Evolving trends in urological claims and damages paid

**Fig. 1:**
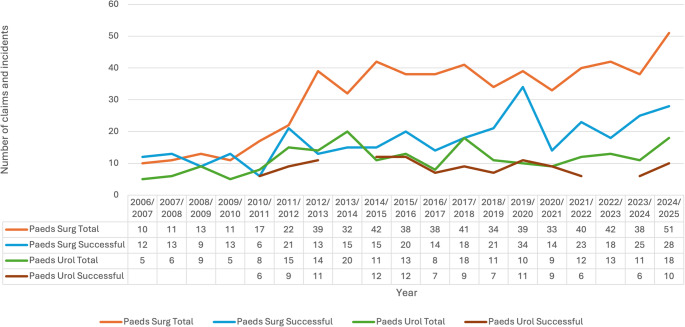
Total number of claims and successful claims for Paediatric Urology and Paediatric Surgery in the last 19 years. *Paeds Surg* paediatric surgery; *Paeds Urol* paediatric urology. Data for selected years were masked by NHS Resolution in accordance with disclosure control policies for small-number categories

The yearly distribution of Paediatric Urology and Paediatric Surgery claims, including those that were successful, is presented in Fig. 1.

Across the 19-year-study period, Paediatric Urology claims increased from 5 in 2006/2007 to 18 in 2024/2025. The steepest yearly increase was by 10, which occurred from 2016/2017 (8 total claims) to 2017/2018 (18 total claims).

For the same amount of time, the number of successful claims also proportionally increased from fewer than 5 in 2006/2007 to 10 in 2024/2025. The largest increase per year occurred from 2023/2024 (6 claims) to 2024/2025 (10 claims) by 4 successful claims. Although this represents an overall upward trend, the relatively small annual numbers mean that year-to-year fluctuations may appear pronounced and should be interpreted with caution.

**Fig. 2:**
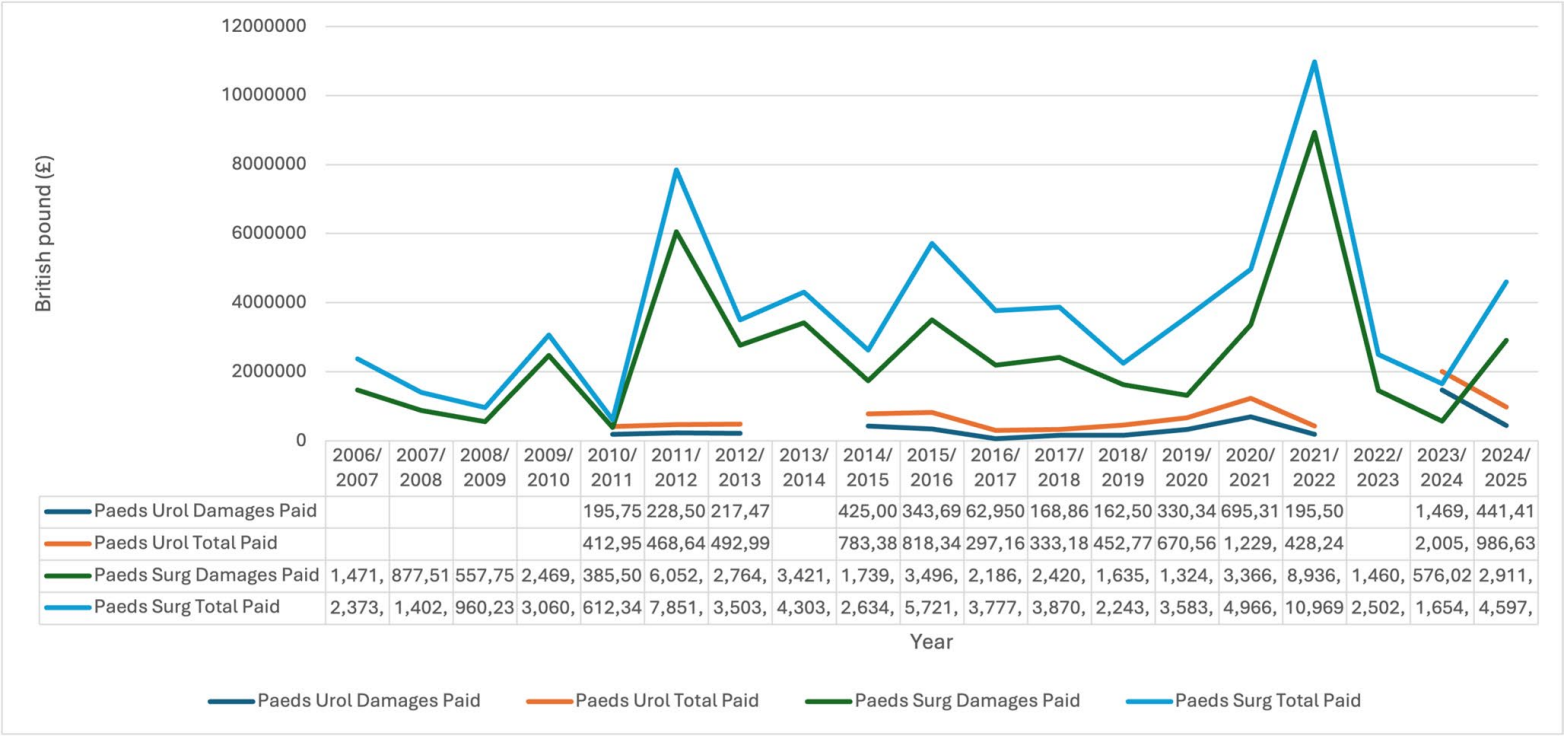
Damages and total amount paid out for successful claims for Paediatric Urology and Paediatric Surgery in the last 19 years. *Paeds Surg* paediatric surgery; *Paeds Urol* paediatric urology. Data for selected years were masked by NHS Resolution in accordance with disclosure control policies for small-number categories

The damages paid out and the total costs including the NHS and claimant legal costs for the past 19 financial years are shown in Fig. 2. The cost associated with damages paid out due to successful claims increased by 2.2-fold from £0.196 million in 2010/2011 to £0.441 million by the end of the 19-year-study period in 2024/2025. Similarly, the total cost of the claims increased by 2.4-fold from £0.412 million in 2010/2011 to £0.986 million in 2024/2025. The data was masked for the period from 2006/2007 to 2009/2010.

### Reasons and primary injuries sustained behind successful urological claims

The leading causes leading to a successful litigation were: failure or delay in diagnosis (29.1%, 39/134), failure or delay in treatment (24.6%, 33/134), inappropriate treatment (4.4%, 6/134), delay in performing an operation (4.4%, 6/134) and failure to organise follow-up arrangements (3.7%, 5/134).

Detailed cause and injury breakdowns were available only for successful claims. Equivalent categorisation was not provided for unsuccessful claims within the dataset.

When grouped, operative causes for successful claims accounted for 9.70% (13/134) and non-operative causes accounted for 66.4% (89/134). Causes masked (< 5) accounted for 32 (23.8%) successful claims. A detailed breakdown of the causes of successful litigation claims for Paediatric Urology over the 19-financial-year period (2006/2007–2024/2025) are presented in Table 1.

Regarding patient harm, the most frequently reported primary injuries associated with successful claims were: removal of testicle (36.6%, 49/134), additional or unnecessary operations (17.1%, 23/134), unnecessary pain (8.9%, 12/134), renal damage or failure (6.7%, 9/134), psychiatric or psychological damage (4.4%, 6/134) and loss of sexual function (3.7%, 5/134). These outcomes are summarised in Table 2.

## Discussion

### Principal findings

The present study presents the first, to the best of our knowledge, comprehensive evaluation of litigation claims within Paediatric Urology in NHS England. Over the study period (2006/2007–2024/2025), both the volume of claims and their associated financial impact increased substantially. Damages rose by approximately 2.2-fold, while overall expenditure increased by 2.4-fold, highlighting the escalating financial burden of litigation on the NHS.

Notably, the predominant drivers of successful claims were delays or failures in diagnosis or treatment, reflecting systemic process-related shortcomings rather than operative errors.

Our findings align with other national reports demonstrating a progressive rise in medicolegal costs across surgical specialties, including adult urology [1, 2, 7]. Previous analyses in adult Urology have shown that most claims were non-operative in nature, findings which were consistent with the present study [1, 2, 7].

Paediatric Urology and Paediatric Surgery differ substantially in case mix and harm phenotype. Paediatric Urology adverse events can involve long term-functional or quality-of-life outcomes such as fertility or urinary function, whereas Paediatric Surgery encompasses a broader spectrum including life-threatening or acute pathology. Therefore, comparisons of claim frequency or cost between specialties should not be interpreted as a proxy for relative clinical severity.

The ratio of Paediatric Urology to Paediatric Surgery claims was approximately 5:10 (0.5) in 2006/2007, rising to 18:51 (0.35) by 2024/2025 at the end of the study period, suggesting that Paediatric Urology claims have not grown disproportionately relative to Paediatric Surgery; if anything, Paediatric Surgery claims have increased at a faster rate. However, given the small annual numbers in both specialties, this ratio should be interpreted with caution.

Claims classified under Paediatric Urology may include acute presentations initially managed by non-urological clinicians but coded under Paediatric Urology within NHS Resolution’s internal database. Therefore, findings may reflect litigation relating to paediatric genitourinary conditions rather than exclusively subspecialist Paediatric Urology practice.

Service configuration warrants explicit consideration. Within NHS England’s hub-and-spoke model, some paediatric urological procedures are delivered in outreach settings by general paediatric surgeons, adult urologists, or general surgeons at district general hospitals, where subspecialist support may not be immediately available. Time-critical acute scrotal presentations, most notably testicular torsion, are frequently managed by adult urologists or on-call surgical teams when transfer to a specialist children’s hospital would introduce harmful delay. These service-configuration effects cannot be disentangled from the present dataset but represent an important contextual factor when interpreting the findings.

Direct comparison with adult Urology litigation trends in NHS England is possible through reference to previously published analyses [1, 2, 7]. Given that adult urology volumes substantially exceed paediatric volumes, any graphical comparison would require indexing by activity or using ratios; such data were not available within the present FOI extract.

### Interpretation and implications

The predominance of non-operative claims indicates organisational and system-level factors constitute the biggest medicolegal challenge within Paediatric Urology. Delays or failures in diagnosis or treatment were the most frequent contributors to successful claims. The introduction of the NHS 18-week referral-to-treatment target has placed additional demands on services, and meeting these standards will require both operational and workforce redesign [8]. Proposed strategies include digitalisation and shifting care from hospitals to the community in line with the NHS 10-year plan [9].

It should be notes that the present analysis lacks a denominator of total paediatric urological activity volume or procedure numbers over the study period. Whether the observed increase in claims is proportional to, or disproportionate relative to, activity growth cannot be determined from the present dataset alone. This represents an important contextual limitation when interpreting apparent trends in litigation risk.

Targeted initiatives such as the Early Notification Scheme, improved access to timely care, consistent adherence to clinical pathways, and robust follow-up processes may help mitigate avoidable harm. Furthermore, improved workforce retention, optimised rota design, and measures to reduce clinician burnout may decrease fatigue-related error [10]. NHS Resolution, in collaboration with the National Audit Office [11] help reduce the financial impact of litigation, enhancing patient safety, and improving transparency on how claims are managed.

It is important to recognise that many of the systemic failures identified such as delayed diagnosis, failure of escalation, and breakdown of follow-up, do not occur in isolation from workforce pressures. Chronic understaffing, rota gaps, and limited access to senior clinical review may potentially create the conditions in which these failures are more likely to occur. Investing in sustainable workforce models, protected supervision capacity, and dedicated follow-up infrastructure may therefore represent some of the most meaningful long-term strategies for reducing litigation burden in paediatric urology.

Although, operative-related claims remain important, they accounted for a smaller proportion of successful claims (9.70%) compared with non-operative causes. Nevertheless, strict adherence to clinical guidelines, safety standards and robust consenting processes remains essential. Ongoing reflective practice, including discussion of adverse events through regular morbidity and mortality meetings, is crucial. The implementation of the Patient Safety Incident Report Framework (PSIRF) further strengthens the NHS’s focus on identifying system-level contributors to harm, aligning closely with the litigation patterns observed in Paediatric Urology and supporting more effective prevention strategies [12].

The exercise of the Duty of Candour and early structured management of complications may also reduce dispute escalation. When adverse outcomes are promptly disclosed, clearly explained, and followed by a structured organisational response, the likelihood of formal litigation may be reduced. Although this dataset cannot capture whether candour was exercised in individual cases, NHS Resolution’s strategic emphasis on early resolution is consistent with the view that transparent communication and prompt acknowledgment of harm are effective upstream mitigators of claims volume and cost [4].

### External influencing

Analysis of financial trends demonstrated a sustained rise in overall expenditure, accompanied by increasing compensation payments. This escalation may be driven largely by a progressive increase in both claimant and NHS legal costs, potentially reflecting shifts within the medicolegal and regulatory environment. It should also be noted that financial values in this analysis were reported in nominal terms and were not adjusted for inflation. Consequently, part of the observed increase in expenditure over time may reflect changes in the value of money rather than solely increased litigation severity. The COVID-19 pandemic represents a significant systemic shock to healthcare delivery in NHS England. Beyond the documented reduction in elective surgical activity during 2020–2021, the pandemic disrupted referral pathways, suspended follow-up services, and delayed investigation and diagnosis, all factors that represent plausible precursors to subsequent litigation. As claim-year does not reliably correspond with to the year of the clinical incident, the full medicolegal impact of the pandemic may not yet be fully apparent in the current dataset, and claims submitted in subsequent years should be monitored accordingly.

The NHS context differs fundamentally from insurance-based systems such as those in the United States, Canada, and much of continental Europe. In insurance-driven systems, litigation may sometimes function as a route through which patients fund ongoing care, whereas in publicly funded systems such as the NHS, compensation fulfils a narrower legal role. Rates and costs of litigation are therefore heavily shaped by the legal framework and compensation mechanisms in operation, and direct cross-system comparisons require harmonised claim definitions and denominators that are not available across national datasets. Published litigation rates from other countries should therefore be interpreted with caution when placed alongside NHS Resolution figures.

### Strengths and limitations of this study

This study represents the first trend analysis using the most recent NHS Resolution dataset spanning a 19-year period and encompassing all Paediatric Urology litigation claims across NHS England, therefore providing a national overview. By stratifying claims according to operative versus non-operative mechanisms and by primary injury, the analysis identifies key high-risk areas in the patient’s care pathway. In addition, detailed reporting of damages payments alongside claimant and NHS legal costs highlights the considerable financial burden imposed on the healthcare system.

There are several limitations. As a retrospective analysis of an administrative database, the findings are reliant on the accuracy and completeness of NHS Resolution records, which are collected for legal rather than research purposes and may be subject to misclassification or coding inaccuracies. Paediatric genitourinary care in NHS England may be delivered by Paediatric Surgery or Adult Urology, therefore genitourinary claims coded under different specialties may not have been captured. Furthermore, the temporal relationship between clinical events and claim resolution is imperfect, as claims are often filed or closed several years after the index incident, potentially obscuring true chronological trends. Finally, disruptions to healthcare delivery during the COVID-19 pandemic including reduced elective activity and delays in investigation are likely to have influenced litigation patterns. However, these effects cannot be reliably quantified using the available data. The dataset by NHS Resolution did not stratify claims by type of urological procedure, urgency (elective or emergency), care setting, or treating specialty. This limits identification of high-risk procedural groups. Therefore, future datasets would benefit from inclusion of procedure category, urgency, care setting, treating specialty, and pathway stage. Such granularity would enable more targeted prevention strategies and would be useful to guide the practicing paediatric urologists.

## Conclusion

Litigation within Paediatric Urology in NHS England has increased substantially over the past 19 years, with rises observed in both total and successful claims. The predominance of non-operative causes (66.41%), indicates delays in diagnosis or treatment and system-level shortcomings, rather than technical operative errors are the principal contributors to litigation in the specialty. Targeted efforts to optimise diagnostic and treatment timelines and pathways, strengthen follow-up systems and improved communication may help reduce the most common sources of litigation. Ongoing monitoring of litigation trends is crucial for identifying emerging risks, supporting the provision of care and service improvement and guiding policies aimed at reducing patient harm and medicolegal exposure across the NHS. The findings should be interpreted in light of the dataset’s inherent limitations, including the absence of procedure-level stratification, activity denominators, and cross-specialty coding completeness. Future datasets that capture procedure category, urgency, care setting, and treating specialty will be essential to enable more targeted and actionable prevention strategies.

**Table 1:** Causes of successful litigation claims for paediatric urology in the last 19 financial years (2006/2007–2024/2025) listed from most to least frequent

Non-operative claims, *n* (%)	
Failure/delay diagnosis	39 (29.1)
Failure/delay treatment	33 (24.6)
Inappropriate treatment	6 (4.4)
Delay in performing operation	6 (4.4)
Fail to follow-up arrangements	5 (3.7)
Failure to warn-informed consent, failure to perform tests, failure to recognise complication of, other, lack of pre-op evaluation, infusion problems, inadequate nursing care, failure to interpret X-Ray, failure to X-Ray, fail antenatal screening, inappropriate discharge	#

Operative claims, n (%)	
Intra-operative problems	8 (5.9)
Failure to perform operation	5 (3.7)
Operator error, performance of operation not indicated, diathermy burns/reaction to prep, failed sterilisation, equipment malfunction, inappropriate case selection,	#

*n* number

**Table 2:** Primary injuries sustained leading to successful litigation claims for Paediatric Urology in the last 19 financial years (2006/2007–2024/2025) listed from most to least frequent

Primary injury, *n* (%)	
Removal of testicle	49 (36.5)
Additional/unnecessary operation(s)	23 (17.1)
Unnecessary pain	12 (8.9)
Renal damage/failure	9 (6.7)
Psychiatric/psychological damage	6 (4.4)
Loss of sexual function	5 (3.7)
Scarring, infertility, fatality, loss of kidney, bladder damage, cosmetic disfigurement, burn(s), other, multiple injuries, incontinence, wrongful birth, reduced life expectancy, brain damage, bruising/extrvasation, other infection	#

*n* number

## Data Availability

No datasets were generated or analysed during the current study.

## Declarations

### Patient and public involvement

Patients and members of the public were not involved in the design, conduct, reporting, or dissemination plans of this research. This study was based on anonymised, aggregated national litigation data obtained from NHS Resolution under the Freedom of Information Act. No individual-level patient data were accessed, and there was no direct patient contact. The research question and outcomes were informed by clinical practice priorities and the need to understand system-level factors contributing to patient harm and medicolegal claims within NHS England.
